# Neutrophil extracellular traps induce aggregation of washed human platelets independently of extracellular DNA and histones

**DOI:** 10.1186/s12964-018-0235-0

**Published:** 2018-05-29

**Authors:** Omar Elaskalani, Norbaini Binti Abdol Razak, Pat Metharom

**Affiliations:** Platelet Research Laboratory, School of Pharmacy and Biomedical Sciences, Curtin Health and Innovation Research Institute, Faculty of Health Sciences, Curtin University, Bentley Campus, Office 160, Building 305, Kent Street, Bentley, Perth, WA 6102 Australia

**Keywords:** Neutrophil, Neutrophil extracellular traps, Platelet, Aggregation, DNA, Histones, Cathepsin G

## Abstract

**Background:**

The release of neutrophil extracellular traps (NETs), a mesh of DNA, histones and neutrophil proteases from neutrophils, was first demonstrated as a host defence against pathogens. Recently it became clear that NETs are also released in pathological conditions. NETs released in the blood can activate thrombosis and initiate a cascade of platelet responses. However, it is not well understood if these responses are mediated through direct or indirect interactions. We investigated whether cell-free NETs can induce aggregation of washed human platelets in vitro and the contribution of NET-derived extracellular DNA and histones to platelet activation response.

**Methods:**

Isolated human neutrophils were stimulated with PMA to produce robust and consistent NETs. Cell-free NETs were isolated and characterised by examining DNA-histone complexes and quantification of neutrophil elastase with ELISA. NETs were incubated with washed human platelets to assess several platelet activation responses. Using pharmacological inhibitors, we explored the role of different NET components, as well as main platelet receptors, and downstream signalling pathways involved in NET-induced platelet aggregation.

**Results:**

Cell-free NETs directly induced dose-dependent platelet aggregation, dense granule secretion and procoagulant phosphatidyl serine exposure on platelets. Surprisingly, we found that inhibition of NET-derived DNA and histones did not affect NET-induced platelet aggregation or activation. We further identified the molecular pathways involved in NET-activated platelets. The most potent single modulator of NET-induced platelet responses included NET-bound cathepsin G, platelet Syk kinase, and P2Y_12_ and αIIbβ3 receptors.

**Conclusions:**

In vitro-generated NETs can directly induce marked aggregation of washed human platelets. Pre-treatment of NETs with DNase or heparin did not reduce NET-induced activation or aggregation of human washed platelets. We further identified the molecular pathways activated in platelets in response to NETs. Taken together, we conclude that targeting certain platelet activation pathways, rather than the NET scaffold, has a more profound reduction on NET-induced platelet aggregation.

**Electronic supplementary material:**

The online version of this article (10.1186/s12964-018-0235-0) contains supplementary material, which is available to authorized users.

## Background

Neutrophils are well known for their crucial role in innate immunity, providing the first line of defence against pathogens through multiple mechanisms [1]. The discovery of a relatively new antimicrobial mechanism, whereby activated neutrophils expel their DNA and proteins forming an extracellular matrix, termed neutrophil extracellular traps (NETs) [2], has gained much interest recently. NETs possess antimicrobial function either by entrapping and immobilising pathogens or presentation of NET-bound antimicrobial proteins [2, 3]. However, NETs can serve as more than just a host defence mechanism, as studies have implicated the role of NETs in inflammatory and autoimmune diseases and pathological conditions including thrombosis [4]. Notably, as the role of NETs in thrombosis is being investigated extensively in recent times, there is potential for NETs to not only serve as therapeutical target for thrombotic diseases but several other clinical conditions such as diabetes, systemic lupus erythematosus, pre-eclampsia and certain types of cancers, all which are known to be associated with increased risk of thrombosis [5–8].

Increasing number of studies are recognising NETs as a procoagulant surface, which is capable of promoting thrombosis both in vitro and in animal models of deep vein thrombosis and arterial thrombosis [9–13]. The NET structure can serve as a scaffold for platelet adhesion and aggregation [9, 14] thus providing a platform for the subsequent formation of thrombi. Furthermore, NETs have been shown to directly promote the activation of intrinsic coagulation pathway leading to thrombin generation [15]. Besides intact NETs being capable of activating coagulation, several components within the NET structure have been reported to activate platelets and initiate or promote coagulation. Cell-free DNA, which makes up the major backbone of NETs, has previously been shown to activate thrombin generation via the intrinsic pathway of coagulation [16]. The second most abundant constituent and protein found on NETs, extracellular histones, have been studied extensively and known to activate platelets and promote coagulation through multiple mechanisms [9, 17–19]. For example, histones are capable of generating thrombin in the presence of plasma and activating platelet aggregation which has been suggested to be mediated through toll-like receptor (TLR) 2 and TLR 4 [18]. However, the involvement of TLRs in platelet aggregation is not clear as Clark et al. have shown that LPS induced platelet activation but not aggregation [20] Furthermore, neutrophil granular enzymes that are bound to NETs such as neutrophil elastase (NE) and cathepsin G (Cat G), can separately promote coagulation and thrombus growth by facilitating intravascular fibrin formation and degrading tissue factor pathway inhibitor [21], while myeloperoxidase (MPO) can prime platelets [22].

Collectively, NETs are a potentially potent agonist of platelet activation and promoter of coagulation, thereby amplifying and supporting thrombus formation. However, despite many studies reporting NETs as promoters of thrombosis, these studies were conducted in whole blood assays or in the presence of plasma, implicating a role of plasma coagulation factors. Thus the capacity of intact cell-free NETs to directly activate washed platelets is not clearly understood. In this study, we investigated the effect of NETs on platelet function including aggregation, secretion, and surface expression of receptors. We also begin to determine molecular mediators and signalling pathways by examining the effect of antagonists of specific NET components and antiplatelet drugs, on the impact of NETs on platelet activation.

## Methods

### Materials

Purified anti-human TLR2, TLR4 blocking antibodies and their matching isotype controls were obtained from BioLegend, Inc., USA. Bay 61–3606 and Phorbol 12-myristate 13-acetate (PMA), ticagrelor and Cell Detection ELISA PLUS kit were from Sigma-Aldrich, Australia. ML-171, aspirin, RGDS, cathepsin G inhibitor I, neutrophil elastase inhibitor (1-(3-methylbenzoyl)-1H-indazole-3-carbonitrile), myeloperoxidase inhibitor 1 (4-Aminobenzoic acid hydrazide), losartan were obtained from Cayman Chemical, USA. Abciximab (ReoPro) and low molecular weight heparin (Clexane enoxaparin sodium) were from Eli Lilly and Sanofi Aventis Australia Pty Ltd., respectively. DNAse I solution was purchased from STEMCELL Technologies Australia Pty Ltd. Collagen and thrombin were from Chrono-log Corporation, USA. Human PMN Elastase ELISA kit was obtained from Abcam Biotechnology, Cambridge, UK.

### Preparation of washed human platelets

Blood was drawn from healthy volunteers into a syringe containing acid-citrate-dextrose (ACD; 1:7 (*v*/v) with informed consent in concordance with the Curtin University Human Research Ethics Committee (approval number HR54/2014). Blood donors were medication-free 2 weeks prior to the day of donation. Washed platelets were prepared, with some modifications, as previously described [23, 24]. Briefly, blood was centrifuged at 150 x *g* for 20 min. Platelet-rich plasma (PRP) was collected and centrifuged at 800 x *g* for 10 min in the presence of 1 μM prostaglandin E1 (PGE1; Cayman Chemical). Platelets were then washed three times in CGS buffer (14.7 mM trisodium citrate, 33.33 mM glucose and 123.2 mM NaCl, pH 7), in the presence of PGE1 (1 μM). Platelets were adjusted to 1 × 10^9^/mL with calcium-free Tyrode-Hepes buffer (5 mM HEPES, 5.5 mM glucose, 138 mM NaCl, 12 mM NaHCO_3_, 0.49 mM MgCl_2_, 2.6 mM KCl, 0.36 mM NaH_2_PO_4_, pH 7.4). Platelets were supplemented with 1.8 mM CaCl_2_ (final concentration) prior to experimentation.

### Preparation of neutrophils and cell-free neutrophil extracellular traps (NETs)

Neutrophils were isolated from human blood using PolymorphPrep (Axis-Shield, Norway), with minor changes to the manufacturer’s protocol. Briefly, blood anticoagulated with EDTA (2 mM) was layered over PolymorphPrep and centrifuged at 500 x *g* for 40 min. The neutrophil fraction was collected and washed twice at 4 °C in Hank’s buffered saline solution (without calcium or magnesium) and resuspended in X-VIVO 15 media (Lonza, Switzerland). Neutrophil purity was > 95% as determined with a haematology analyser (Mindray, BC-VET2800). Cell-free NETs were isolated as previously described [25] with minor changes to the protocol. This method of NET isolation does not involve using DNase or EDTA [26], which may confound platelet response to NETs. Briefly, neutrophils (2.5 × 10^6^/mL) were stimulated with 500 nM PMA for 3 h at 37 °C and 5% CO_2_. The supernatant, containing PMA, was discarded and the NET monolayer was detached with phosphate-buffered saline (PBS). The cell debris was pelleted by centrifugation at 480 x *g* for 10 min at 4 °C. The supernatant was further centrifuged at 15,000 x *g* for 20 min at 4 °C to pellet DNA then resuspended in PBS at 100 μl per 1 × 10^7^ of stimulated neutrophils to obtain cell-free NETs. Cell-free NETs were characterised by detecting DNA-histone complex and neutrophil elastase using Cell Detection ELISA PLUS kit (Sigma Aldrich) and Human PMN Elastase ELISA kit (Abcam), respectively. Cell-free NETs were incubated with platelets at 10% of final reaction volume (i.e. 1-volume NET solution to 9-volume platelets).

### Platelet aggregation assay

Washed platelets (3 × 10^8^/mL) in Tyrode-HEPES buffer supplemented with 1.8 mM calcium chloride were incubated in the presence of cell-free NETs (10% of final reaction volume) and platelet aggregation was monitored at 37 °C with continuous stirring at 1200 rpm in a light transmission aggregometer (Model 700 Aggregometer, Chrono-log Corporation, USA) for at least 20 min. Tyrode-HEPES buffer was used as a blank. Where inhibitors were used, platelets were pre-incubated for 15 min at 37 °C prior to incubation with NETs. Control samples were incubated with the corresponding volume of buffer.

### Platelet-dense granule secretion assay

Platelet secretion was determined by measuring ATP release using luciferin/luciferase reagent (Chrono-Lume, Chrono-log Corporation, USA). Briefly, 90 μl of platelets (1 × 10^8^/mL) in Tyrodes-HEPES buffer (with calcium) were incubated with 10 μl of NETs with gentle shake at 37 °C for 1 and 10 min before adding 5 μl of Chrono-Lume reagent. The luminescence was measured using Enspire Multimode Plate Reader (PerkinElmer, USA). Where anti-platelet drugs were used, platelets were pre-incubated with the drugs for 15 min at 37 °C before incubating with NETs.

### P-selectin exposure and αIIbβ3 activation

Platelet activation was measured by detecting P-selectin and active-form αIIbβ3 on the platelet surface using flow cytometry. Where inhibitors were used, platelets were pre-incubated for 15 min at 37 °C before adding NETs. Whenever inhibitors of components of NETs (i.e. DNAse I, cathepsin G, myeloperoxidase, and elastase inhibitors) were used, NETs were pre-incubated for 30 min at 37 °C. The specificity of inhibitors used was also examined for their effect on thrombin (0.1 U/mL) and collagen (5 μg/mL)–induced platelet activation. Inhibitor-, or vehicle-, treated washed human platelets (1 × 10^8^/mL) were treated with NETs (10% of final volume) and stained with phycoerythrin-conjugated mouse anti-human CD62P (P-selectin) and fluorescein isothiocyanate-conjugated mouse anti-human PAC1 (active-form αIIbβ3), or suitable isotype control antibodies for 15 min in the dark. All antibodies were from BD Biosciences. Samples were analysed by flow cytometry (BD LSRFortessa™ cell analyzer).

### Phosphatidylserine (PS) exposure

Platelets (3 × 10^8^/mL) were incubated with NETs for 30 min at 37 °C with continuous stirring at 1200 rpm. Whenever inhibitors were used, NETs were pre-incubated for 30 min at 37 °C. Thrombin (0.1 U/mL) was used as positive control. Platelets were then stained with Annexin V-FITC (BioLegend, USA) in binding buffer according to manufacturer’s instructions for 15 min in the dark. Samples were then washed in binding buffer and analysed by flow cytometry (BD LSRFortessa™ cell analyzer).

### Antibodies and western blot

Rabbit antibodies specific for p-Akt (Ser473), p-Erk1/2 (Thr202/Tyr204), p-Syk (Tyr352), p-Tyr1000 and α-actinin were obtained from Cell Signalling Technology (USA). Platelets (3 × 10^8^/mL) were incubated with NETs (10% of final reaction volume) at 37 °C for 3 min with continuous stirring at 1200 rpm. Platelets were then lysed in Laemmli sample buffer supplemented with Protease/Phosphatase Inhibitor Cocktail (Cell Signalling Technology) and β-mercaptoethanol. Forty-five μl of protein sample was loaded per lane and separated by sodium dodecyl sulphate–polyacrylamide gel electrophoresis then transferred to a polyvinylidene difluoride (PVDF) protein blotting membrane with 0.2 μm pore size (GE Healthcare Life Sciences). The PVDF membrane was blocked in 5% non-fat powdered milk in Tris-buffered saline with 0.1% Tween 20 (or 3% bovine serum albumin (BSA, Bovogen Biologicals Pty Ltd., Australia) in TBS-T for detection of p-Tyr-1000) at room temperature for 1 h. After a brief rinse with TBS-T, the membrane was incubated overnight at 4 °C with primary antibodies at 1:1000 dilution. The primary antibody was detected with secondary horseradish peroxidase-conjugated anti-rabbit antibody (Jackson Immune Research, USA) at 1:40000 dilution. The membrane was developed using Amersham ECL Prime Western Blotting Detection Reagent (GE Healthcare Life Sciences, USA), and chemiluminescence was detected using ChemiDoc imaging system (Bio-Rad, USA).

### Statistical analysis

Data were analysed using GraphPad PRISM 4.0 software. Results are expressed as the mean ± standard error (SEM). One-way ANOVA with posthoc Bonferroni’s Multiple Comparison Test were used to examine the statistical significance between means. Differences were considered significant at *P* < 0.05.

## Results

### NETs induce aggregation, secretion and activation of washed human platelets

**Fig. 1:**
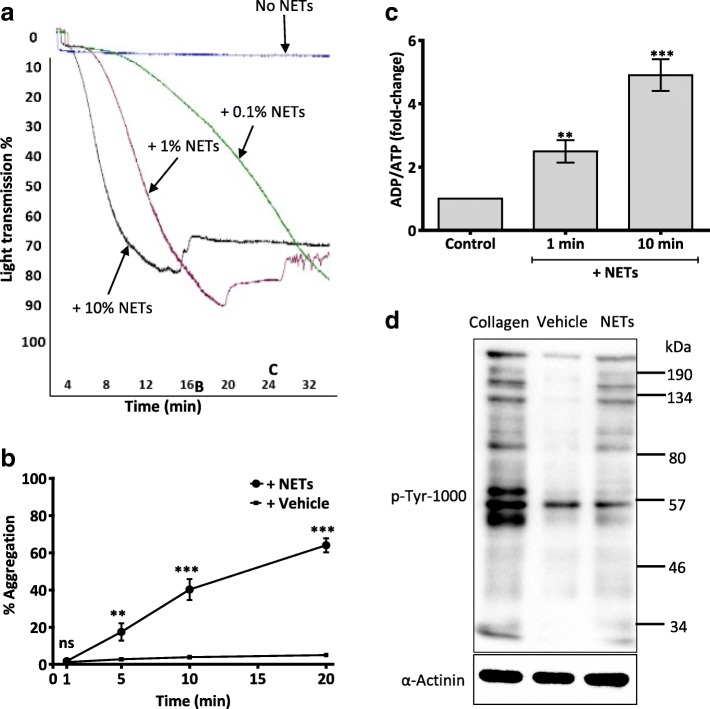
NETs directly induce aggregation of washed human platelets. **a** Representative traces showing dose-dependent aggregation of washed human platelets (WP, 3 × 10^8^ /mL) in response to cell free-NETs. 0.1, 1 and 10% NETs refers to NETs constituting 0.1, 1 or 10% of final reaction volume, respectively (e.g. 10% NET is 1-volume NET solution to 9-volume platelets). Platelet aggregation was measured by light transmission aggregometer (Chrono-log). NETs were used at 10% of final reaction volume for following experiments*. **b** Progress time curve displaying % of NET-induced platelet aggregation which steadily increased over 20 min. **c** ATP/ADP release from NET-stimulated platelets. WP (1 × 10^8^/mL) were incubated with NETs with a gentle shake at 37 °C for 1 and 10 min before adding Chrono-Lume reagent. Luminescence was measured using Enspire Multimode Plate Reader (PerkinElmer). NETs induced significant ATP/ADP secretion from platelets at 1 and 10 min. Results represent fold change in luminescence arbitrary absorbance unit relative to vehicle (PBS) control. Data expressed as mean ± SEM; **P < 0.01, ***P < 0.0001, *N* ≥ 5. **d** Platelet signalling in response to NETs was examined by Western blot analysis. WP (3 × 10^8^/mL) were incubated with collagen (5 μg/mL), vehicle (PBS) or NETs for 3 min. Twenty μl of total cell lysate was analysed by sodium dodecyl sulphate–polyacrylamide gel electrophoresis and immunoblotted for phospho-Tyrosine (p-Tyr-1000). Equal loading was verified by α-actinin. Western blots indicate tyrosine-phosphorylated proteins in NET-stimulated platelets. *10% NET solution is prepared from 1 × 10^7^/ml of PMA-activated neutrophils, however due to losses during preparation steps, 10% NET is estimated to contain histone/DNA complexes equivalent to 3.8 ± 1.2 × 10^6^/mL neutrophils (Additional file 1: Figure S1). The elastase content present in a 10% NET reaction volume is 292 ± 172 pg/mL

We first examined the ability of cell-free NETs to directly induce aggregation of washed human platelets independently of the coagulation pathway. Platelets were washed three times in CGS buffer to remove any contaminating plasma, then resuspended in Tyrode-HEPES buffer (with 1.8 mM calcium chloride). Light transmission aggregometry was used to measure platelet aggregation. Autologous cell-free NETs at different dilutions induced marked platelet aggregation (Fig. 1a). Interestingly, platelet aggregation response was apparent only after 5 mins in the presence of the highest concentration of NETs used (10% of final reaction volume) (Fig. 1b). For all experiments, it was imperative that NETs were used within the same day of isolation as freezing or storing NETs at 4 °C for more than 24 h completely abolished their activity.

As platelet secretion amplifies platelet aggregation, we assessed the ability of NETs to induce platelet dense granule secretion of ATP/ADP using a luminescence assay. NETs triggered significant ATP/ADP secretion from platelet dense granules compared to vehicle control (Fig. 1c). Platelet secretion was examined after 1 and 10 min incubation with NETs to identify the time required for secretion compared to aggregation. NETs induced significant platelet secretion by 1 min, while aggregation did not occur until 5 min (Fig. 1b-c), indicating that NET-induced platelet secretion precedes aggregation. Furthermore, we also examined if platelet functional response occurred concomitantly with intracellular signalling, particularly the phosphorylation of proteins at tyrosine residues. Indeed, NETs induced tyrosine phosphorylation of several substrates with strong migrating bands at ~ 134, 80 and 60 kDa compared to vehicle control (Fig. 1d).

### NETs induce surface expression of receptors and phosphatidyl serine exposure on platelets

**Fig. 2:**
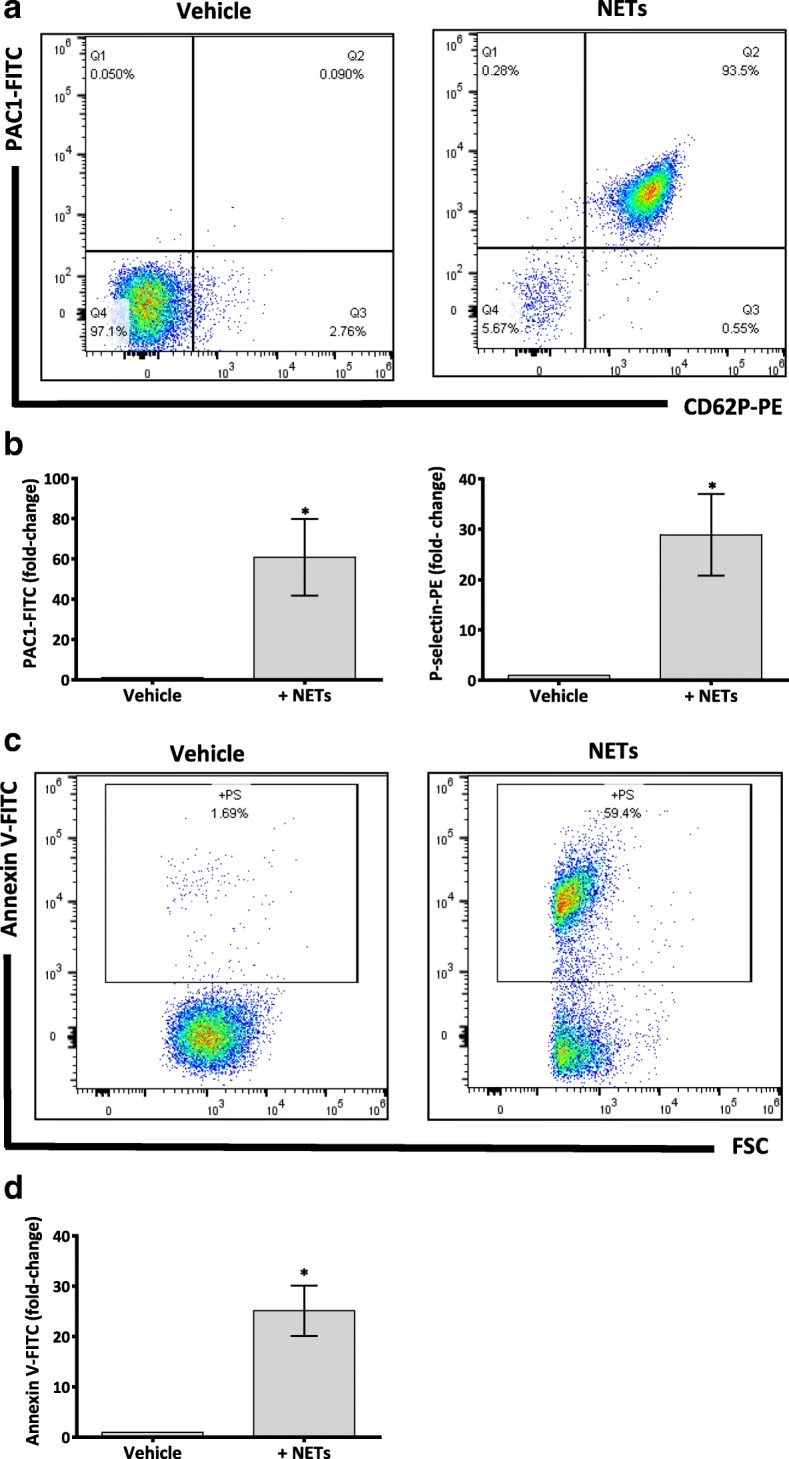
NETs induce platelet activation and phosphatidyl serine (PS) exposure on washed human platelets. **a** Representative dot plot of CD62P (P-selectin), PAC1 (active-form αIIbβ3) fluorescence. WP (1 × 10^8^/mL) were treated with vehicle (PBS) or NETs then incubated with labelled monoclonal antibodies phycoerythrin (PE)-conjugated CD62P and fluorescein isothiocyanate (FITC)-PAC1 for 10–15 min in the dark. The reaction was stopped by fixing cells in 2% paraformaldehyde before analysing samples with flow cytometry (BD LSRFortessa™ cell analyzer). NETs induced expression of P-selectin and active-form αIIbβ3. **b** Fold change in the geometrical mean fluorescence of P-selectin-PE and PAC1-FITC in NET-activated platelets compared to vehicle-treated platelets. **c** WP (3 × 10^8^/mL) were incubated with vehicle (PBS) or NETs for 30 min at 37 °C with continuous stirring at 1200 rpm. PS was detected by incubating platelets with Annexin V-FITC in binding buffer for 15 min in the dark. Samples were then washed in binding buffer and analysed by flow cytometry (BD LSRFortessa). NETs induced PS exposure compared to vehicle control. **d** Fold change in geometrical mean of fluorescence of Annexin V-FITC in NET-activated platelets compared to vehicle-treated platelets. In all assays, NETs constituted 10% of final reaction volume and contains 292 ± 172 pg/mL of NET-elastase. Data are expressed as mean ± SEM,*P < 0.05, *n* ≥ 4

We also examined α-granule secretion by measuring the surface expression of P-selectin. Platelets incubated with NETs for 10 to 15 min showed a significant increase of P-selectin surface expression as detected by flow cytometry (Fig. 2a-b). A conformational change in platelet αIIbβ3 receptor is required for platelet aggregation [27]. Therefore we assessed platelet surface expression of active-form αIIbβ3 using PAC1 monoclonal antibody. As shown in Fig. 2a-b, NETs induced a conformational change of αIIbβ3 from resting to an activated state. As PS exposure on platelets can propagate coagulation [28], we assessed the ability of NETs to induce PS exposure on the platelet surface. Platelets were incubated with NETs for 30 min at 37 °C with continuous stirring at 1200 rpm before analysing PS expression. Annexin V-FITC was used to stain PS and was analysed by flow cytometry. NETs induced a marked increase in PS expression on platelet’s surface (Fig. 2c-d), suggesting that NET-activated platelets can provide a procoagulant surface.

### The role of NET-derived DNA, histones, cat G, and MPO in NET-induced platelet response

As the major components of NETs are DNA and histones, we first investigated whether the effect of NETs on washed platelets is mediated via NET-derived DNA and/or histones. DNA-histone complexes were confirmed in cell-free NETs using Cell Detection ELISA PLUS kit. Based on comparisons between whole neutrophil lysates and NET dilution samples, the concentration of DNA-histone complex in cell-free NETs used in majority of experiments (i.e. 10% final NETs) was equivalent to the amount present in approximately 3.8 ± 1.2 × 10^6^/mL of neutrophil lysate (Additional file 1: Figure S1). Additionally, the 10% cell-free NET solution was determined to contain 292 ± 172 pg/mL of elastase, another major protein component of NETs (Additional file 1: Figure S2).

**Fig. 3:**
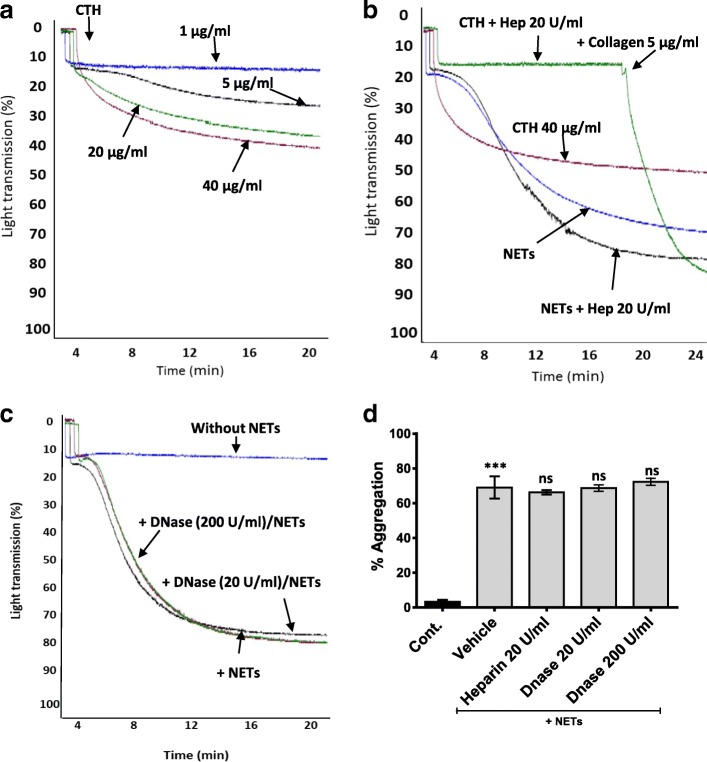
Heparin and DNase did not reduce NET-induced platelet aggregation. **a** Representative traces showing dose-dependent aggregation of washed human platelets (WP, 3 × 10^8^ /mL) in response to calf thymus histones (CTH; 1, 5, 20 and 40 μg/mL). **b** Representative aggregation traces showing the effect of heparin on NET and CTH-induced platelet aggregation. The reactions were allowed to proceed for 18 min, then collagen (5 μg/mL) was added to the CTH-Heparin reaction to test platelet functional response. **c** Representative aggregation traces showing the effect of DNase on NET-induced platelet aggregation. NETs were pre-treated with DNase (20, 200 U/mL) for 30 min at 37 °C before addition to WP (3 × 10^8^/mL). Traces are representative of 3 independent experiments. **d** Bar graphs depict % of platelet aggregation treated with NET alone or with Heparin and DNase. Percentage platelet aggregation was measured at 20 min. In all assays, NETs constituted 10% of final reaction volume and contains 292 ± 172 pg/mL of NET-elastase. Data are expressed as mean ± SEM,*P < .05; ns: non-significant, *n* ≥ 3

Calf thymus histones (CTH) are well-established platelet agonists [29] and were used as a positive control. CTH (1, 5, 20 and 40 μg/mL) induced a dose-dependent aggregation of washed human platelet (Fig. 3a). Heparin can strongly bind to and abate the effect of histones on platelets [30–32]. Indeed, heparin 20 U/mL completely abated CTH but not NET-induced platelet aggregation (Fig. 3 b, d). Collagen (5 μg/mL) was added to the CTH-heparin reaction in order to verify that the platelet functional response to another agonist remained unaffected (Fig. 3b).

In order to test the impact of DNA on NET-induced platelet aggregation, NETs were pre-treated with DNase (20, 200 U/mL) for 30 min at 37 °C before adding to platelets (where NETs was used at 10% of the final reaction volume). DNase can dismantle NET-DNA (Additional file 1: Figure 3). However, DNase did not reduce the ability of NETs to induce aggregation of washed human platelets (Fig. 3c & d), suggesting neither DNA nor histones are major contributors to NET-induced washed platelet aggregation. Since histones can activate platelets, which has suggested to be mediated through toll-like receptor (TLR) 2 and TLR4 [18], platelets were pre-incubated with TLR2- and TLR4-blocking antibodies or matching isotype controls (50 μg/mL) for 15 min before incubation with NETs. Similarly, anti-TLR2 and -TLR4 antibodies also did not affect NET-induced platelet activation (Additional file 1: Figure S4). It was recently reported that calf thymus histones can activate platelets through GPVI receptor [29]. However, pre-incubating platelets with a GPVI inhibitor, losartan (30 μM [33–35]) did not affect NET-induced platelet aggregation (Additional file 1: Figure S5). Our data suggest that neither DNA nor histones contribute to NET-induced washed platelet aggregation or activation.

**Fig. 4:**
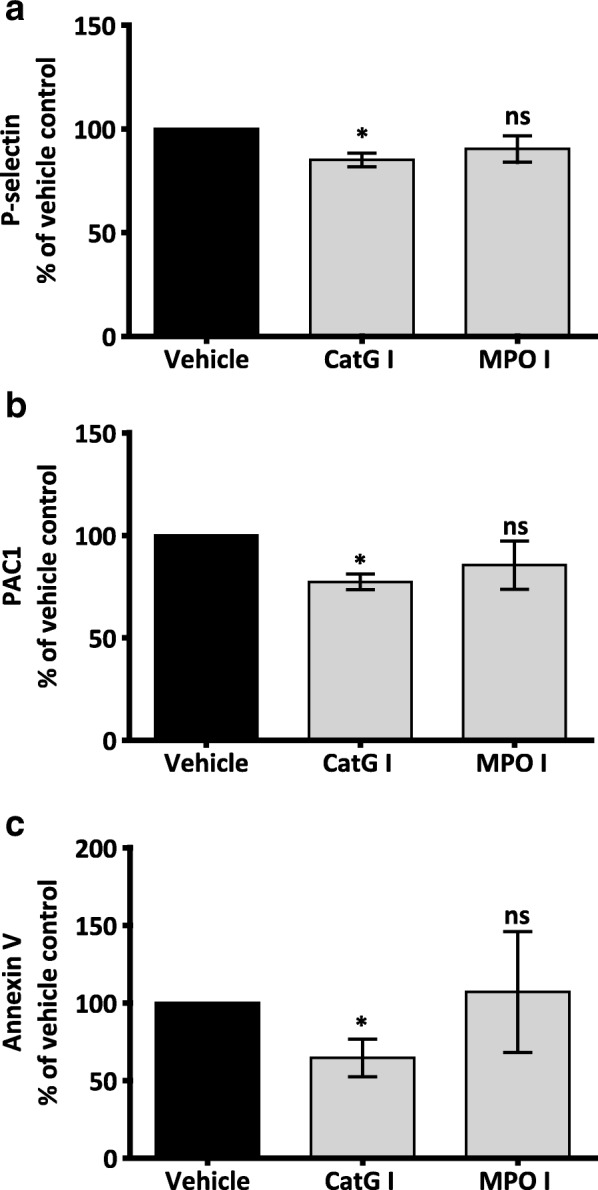
Inhibition of Cat G, but not MPO, attenuates NET-induced platelet response. Flow cytometry analysis of P-selectin, PAC1, and PS exposure on platelets. NETs were pre-treated with vehicle or inhibitors for 30 min at 37 °C. **a-b** WP (1 × 10^8^/mL) were incubated with NETs pre-treated with vehicle (0.1% DMSO in PBS), Cat G I (0.5 μM) or MPO I (50 μM) for 10–15 min in the dark with labelled monoclonal antibodies that detect P-selectin (CD62P) and active αIIbβ3 (PAC1). The reaction was stopped by fixing cells in 2% PFA before analysing samples with flow cytometry (BD LSRFortessa). Bar graphs depict the % inhibition in P-selectin, and active αIIbβ3 expression in platelets treated with NETs pre-treated with different inhibitors compared to platelets treated with NETs that were pre-treated with vehicle. Results were normalized for each donor relative to NET-induced platelet response. **c** WP (3 × 10^8^/mL) were incubated with NETs pre-treated with vehicle (0.1% DMSO in PBS), Cat G I (0.5 μM) or MPO I (50 μM) for 30 min at 37 °C with continuous stirring at 1200 rpm. Platelets were then stained with Annexin V-FITC in binding buffer for 15 min in the dark. Samples were then washed in binding buffer and analysed by flow cytometry (BD LSRFortessa). Bar graph depicts the % inhibition in PS expression in platelets treated with NETs pre-treated with different inhibitors compared to platelets treated with NETs pre-treated with vehicle. Results were normalised for each donor relative to NET-induced platelet response. In all assays, NETs constituted 10% of final reaction volume and contains 292 ± 172 pg/mL of NET-elastase. Data are expressed as mean ± SEM,*P < .05; ns: non-significant, *n* = 4

Extracellular DNA and histones gained considerable interest for their contribution to NET-induced thrombosis, while little attention has been paid to other components of NETs such as neutrophil proteases. Previous studies have reported that Cat G and MPO can modulate platelet response [36, 37]. To determine the effect of NET-derived Cat G and MPO on platelet activation, we used Cat G and MPO inhibitors at concentrations previously described in the literature [36, 37]. Although Cat G inhibition did not significantly affect the maximum aggregation response of platelet to NETs, we observed a trend of increased aggregation lag time (data not shown). Interestingly, Cat G inhibitor (Cat G I) but not MPO inhibitor (MPO I) significantly reduced NET-induced platelet expression of P-selectin (% Max response: 100 vs. 85 ± 3.2; *P < 0.05, *n* = 4), PAC1 (% Max response: 100 vs. 77.3 ± 3.7; *P < 0.05, n = 4) and PS exposure (% Max response: 100 vs. 64.6 ± 12.1; *P < 0.05, *n* = 4) (Fig. 4a-c). The concentration of Cat G I used (0.5 μM) did not affect platelet physiological response to thrombin (0.1 U/mL) (Additional file 1: Figure S6), confirming that the inhibitory response is specific to NET-bound Cat G activation of platelets. Additionally, similarly to MPO, neutrophil elastase did not markedly affect NET-induced platelet activation (Additional file 1: Figure S7).

### SYK and NOX1 contribute to NET-induced platelet response

**Fig. 5:**
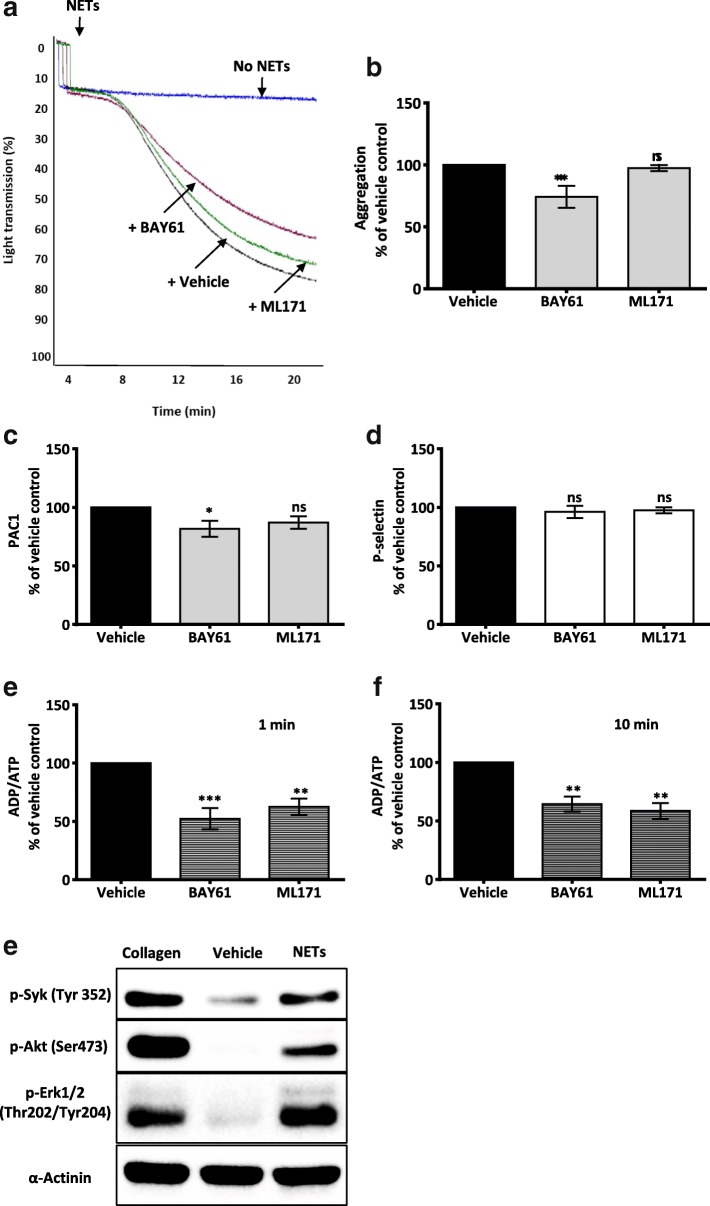
Syk, but not NOX1, inhibition attenuated NET-induced platelet aggregation. **a** Representative aggregation traces showing the effect of Syk phosphorylation (BAY61, 5 μM) and NOX1 (ML171, 5 μM) inhibitors on NET-induced platelet aggregation. WP (3 × 10^8^/mL) were pre-incubated with the inhibitors for 15 min at 37 °C before addition of NETs. Platelet aggregation was measured using transmission aggregometer (Chrono-log). **b** Bar graph comparing the effect of BAY61 and ML171 on NET-induced platelet aggregation. Platelet aggregation percentage was calculated after 20 min on a light transmission aggregometer. Results were normalized for each donor relative to NET-induced platelet aggregation. Data are expressed as mean ± SEM, ***P < .001; ns: non-significant, *n* = 7. (**c-d**) Bar graphs comparing the effect of BAY61 (5 μM) and ML171 (5 μM) on platelet activation (measured by P-selectin and active αIIbβ3 expression, *n* = 3) and (**e-f**) ATP/ADP secretion (*n* = 6) elicited by NETs. Results were normalized for each donor relative to NET-induced platelet response. Data are expressed as mean ± SEM, *P < 0.05, **P < 0.01, ***P < 0.001, ns: non-significant. **e** Syk phosphorylation is augmented by NETs. WP (3 × 10^8^/mL) were incubated with collagen (5 μg/mL), vehicle (PBS) or NETs for 3 min. Forty-five μl of total cell lysate was then analysed by sodium dodecyl sulphate–polyacrylamide gel electrophoresis and immunoblotted for p-Syk, p-Akt and p-Erk1/2. Equal loading was verified by α-actinin. In all assays, NETs constituted 10% of final reaction volume and contains 292 ± 172 pg/mL of NET-elastase. The immunoblots are representative sample of 3 independent experiments

We began to delineate the platelet receptors and downstream pathway involved in NET-induced platelet activation. The non-receptor tyrosine kinase Syk mediates signalling from major platelet receptors, including the histone receptors GPVI and TLR2 [29]. Whereas NADPH oxidase (NOX) regulates GPVI-induced reactive oxygen species generation and subsequent thromboxane A2 (TxA2) production [38]. We demonstrate that platelet Syk phosphorylation was augmented upon exposure to NETs, which was accompanied by upregulation of the downstream signalling molecules p-Akt and p-Erk1/2 (Fig.5e). A Syk phosphorylation inhibitor (Bay 61–3606, 5 μM) reduced NET-induced platelet aggregation (% Max Agg: 100 vs. 74.21 ± 8.8; ***P < 0.001, *n* = 7) (Fig. 5b), dense granule secretion (% Max secretion at 1 min: 100 vs. 52.2 ± 9.2, ***P < 0.001; at 10 min: 100 vs. 64.4 ± 6.3; **P < 0.01, *n* = 6) (Fig. 5e-f), and PAC1 expression (% Max response: 100 vs. 81.7 ± 6.9; *P < 0.05, *n* = 3) (Fig. 5c), while P-selectin expression remained unchanged (Fig. 5d). On the other hand, NOX1 inhibitor (ML171, 5 μM) did not alter NET-induced platelet aggregation or activation (P-selectin and active-form αIIbβ3 expression) (Fig. 5a-d), however, it significantly reduced NET-induced platelet dense granule secretion (% Max secretion at 1 min: 100 vs. 62.4 ± 7.1; **P < 0.01; at 10 min: 100 vs. 58.6 ± 6.6; **P < 0.01, *n* = 6) (Fig. 5d). Collectively, these results highlight the diversity of platelet pathways that are activated by NETs.

### P2Y_12_ but not cyclooxygenase pathway is required for NET-induced platelet aggregation

**Fig. 6:**
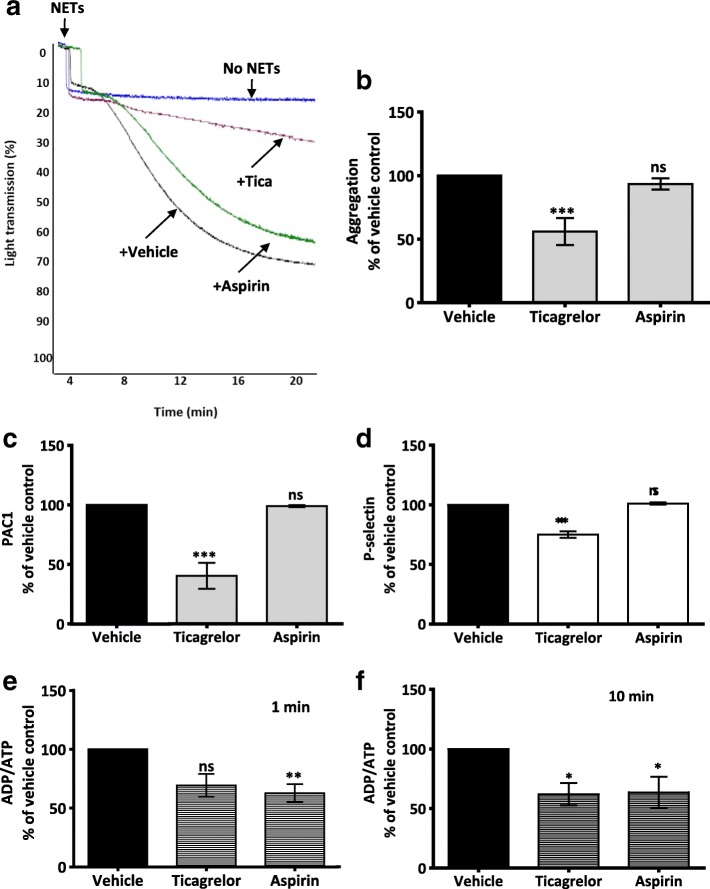
Inhibition of cyclooxygenase pathway in platelets attenuated NET-induced platelet secretion but not aggregation, while inhibition of P2Y_12_ affects both. **a** Representative aggregation traces showing the effect of ticagrelor (1 μM) and aspirin (100 μM) on NET-induced platelet aggregation. WP (3 × 10^8^/mL) were pre-incubated with the inhibitors for 15 min at 37 °C before addition of NETs. Platelet aggregation was measured by light transmission aggregometry (Chrono-log). **b** Bar graph comparing the effect of ticagrelor (1 μM) and aspirin (100 μM) on NET-induced platelet aggregation. Platelet aggregation percentage was calculated after 20 min on a light transmission aggregometer. Results were normalized for each donor relative to NET-induced platelet aggregation. Data are expressed as mean ± SEM, ***P < .001; ns: non-significant, *n* = 3. (**c-d**) Bar graphs comparing the effect of ticagrelor (1 μM) and aspirin (100 μM) on platelet activation (measured by P-selectin and active αIIbβ3 expression) (*n* = 3) and (**e-f**) ATP/ADP secretion (*n* = 5) elicited by NETs. In all assays, NETs constituted 10% of final reaction volume and contains 292 ± 172 pg/mL of NET-elastase. Results were normalized for each donor relative to NET-induced platelet response. Data are expressed as mean ± SEM, *P < 0.05; **P < 0.01; ***P < 0.001, ns: non-significant

Drugs that target either P2Y_12_ (e.g., ticagrelor) or cyclooxygenase pathway (aspirin) are clinically available and crucial in the management of thrombosis [39]. Therefore, we were interested in investigating their effect on NET-induced platelet response. Ticagrelor markedly reduced NET-induced platelet aggregation (% Max Agg: 100 vs. 56 ± 10.1; ***P < 0.001, *n* = 5) (Fig. 6b), dense granule secretion (% Max secretion; at 1 min: 100 vs. 69.3 ± 9.8; *P* = 0.052; at 10 min: 100 vs. 62.1 ± 9.4; *P < 0.05, *n* = 5) (Fig. 6e-f), and expression of P-selectin (% Max response: 100 vs. 72.9 ± 2.9; ***P < 0.001, *n* = 3) (Fig. 6d) and PAC1 (% Max response: 100 vs. 40.34 ± 11; ***P < 0.001, n = 3) (Fig. 6c). Surprisingly, aspirin did not alter NET-induced platelet aggregation or expression of P-selectin and PAC1 (Fig. 6b-d, Additional file 1: Figure S8), however it significantly reduced NET-induced platelet dense granule secretion (% Max secretion; at 1 min: 100 vs. 62.6 ± 7.8; **P < 0.01, *n* = 5; at 10 min: 100 vs. 63.5 ± 13.1; *P < 0.05, *n* = 5) (Fig. 6e-f). These findings suggest a broader role of ticagrelor, but not aspirin, in reducing NET-induced platelet response.

### NET-induced platelet response is dependent on integrin αIIbβ3

**Fig. 7:**
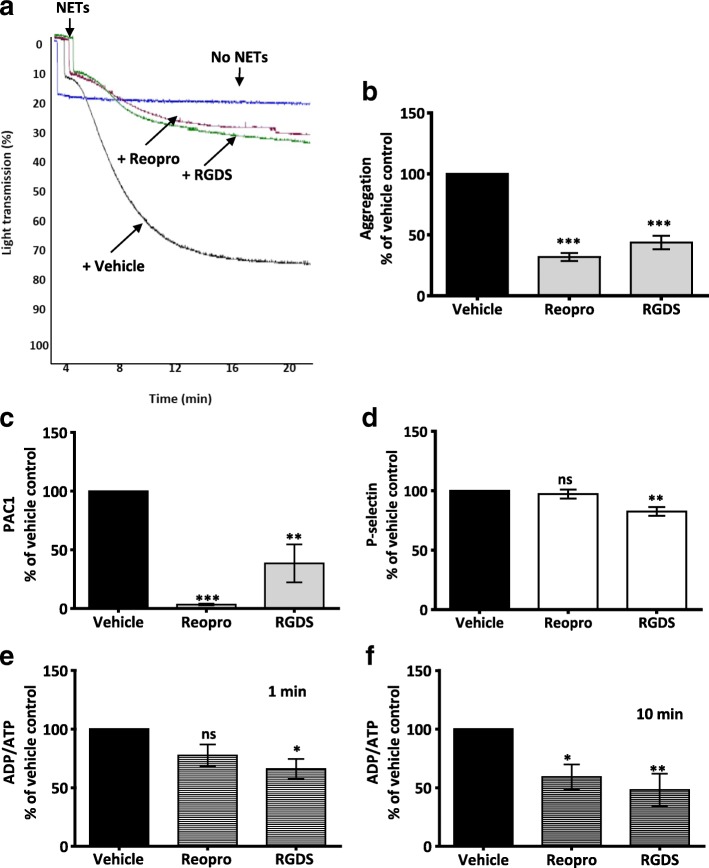
Inhibition of αIIbβ3 attenuates NET-induced platelet response. **a** Representative aggregation traces showing the effect of Reopro (25 μg/mL) and RGDS (100 μM) on NET-induced platelet aggregation. WP (3 × 10^8^/mL) were pre-incubated with the inhibitors for 15 min at 37 °C before addition of NETs. Both Reopro and RGDS reduced platelet aggregation. Platelet aggregation was measured by light transmission aggregometry (Chrono-log). **b** Bar graph comparing the effect of Reopro (25 μg/mL) and RGDS (100 μM) on NET-induced platelet aggregation. Platelet aggregation percentage was calculated after 20 min on a light transmission aggregometer. Results were normalised for each donor relative to NET-induced platelet aggregation. Data are expressed as mean ± SEM,***P < .001. **c-f** Bar graphs comparing the effect of Reopro (25 μg/mL) and RGDS (100 μM) on platelet activation (measured by P-selectin and active αIIbβ3 expression, *n* = 3) and ATP/ADP secretion (*n* = 5) elicited by NETs. In all assays, NETs constituted 10% of final reaction volume and contains 292 ± 172 pg/mL of NET-elastase. Results were normalised for each donor relative to NET-induced platelet response. Data are expressed as mean ± SEM, *P < 0.05; **P < 0.01, ns: non-significant

Considering the role of NETs in mediating platelet adhesion and spreading [30], we were interested in examining the effect of platelet adhesion receptor αIIbβ3 in NET-induced platelet response. Reopro, a monoclonal antibody that binds to and inhibits the active form of αIIbβ3, dramatically reduced NET-induced platelet aggregation (% Max Agg: 100 vs. 31.2 ± 3.3; ***P < 0.001, *n* = 4) (Fig. 7b) and dense granule secretion (% Max secretion; at 1 min: 100 vs. 50 ± 12.2; *P* = 0.075; at 10 min: 100 vs. 59.1 ± 10.8; *P < 0.05, *n* = 5) (Fig. 7e-f). Moreover, Reopro completely inhibited NET-induced PAC1 expression (Fig. 7c), most likely due to competitive binding to αIIbβ3 which is also the target for the PAC1 antibody. However, Reopro did not reduce NET-induced platelet P-selectin expression (Fig. 7d), suggesting that NETs do not trigger αIIbβ3 outside-in signalling in platelets.

RGDS (100 μM), a peptide that binds to αIIbβ3 and prevents conformational change triggered by inside-out signalling, significantly reduced NET-induced platelet aggregation (% Max Agg: 100 vs. 43.7 ± 5.6; ***P < 0.001, *n* = 3) (Fig. 7b) and dense granule secretion (% Max secretion; at 1 min: 100 vs. 66 ± 8.7; *P < 0.05; at 10 min: 100 vs. 48.2 ± 13.8; **P < 0.01, *n* = 5) (Fig. 7e-f). Platelet activation show that RGDS markedly reduced NET-induced platelet P-selectin expression (% Max response: 100 vs. 82.6 ± 3.7; **P < 0.01, *n* = 3) (Fig. 7d) and PAC1 expression (% Max response: 100 vs. 38.4 ± 16.2; **P < 0.01, *n* = 3) (Fig. 7c). Overall, our results confirm the crucial role of αIIbβ3 in NET-induced platelet response.

## Discussion

Our study explored the effect of in vitro-generated NETs on washed human platelets. As described in the methods, cell-free NETs were isolated from PMA-activated human neutrophils. PMA is a known platelet agonist [40], however PMA was washed out with the culture media after 3 h incubation with neutrophils, thus it is highly unlikely that NET-induced platelet aggregation was confounded by PMA. Unlike the widely used method of cell-free NET preparation by Urban et al., [26], we did not use this method involving DNase/EDTA, as EDTA can hinder platelet functional response [41]. We demonstrate that intact cell-free NETs exhibit the capacity to directly activate several platelet responses, such as aggregation, dense and α-granule secretion (ADP release and P-selectin expression), PS exposure and activation of integrin αIIbβ3, which occurred independently of the presence of coagulation factors or thrombin. NETs triggered a dose-dependent aggregation response in platelets with delayed lag time which correlates with the ability of NETs to first induce rapid platelet dense granule secretion.

In addition to being a procoagulant platform, NETs induced PS exposure on platelet’s surface, a characteristic feature of procoagulant platelets. In the presence of small amounts of activated coagulation factors, PS can instigate thrombin generation, which can directly activate platelets and conversion of fibrinogen to fibrin [42]. In washed platelets, strong or multiple agonists can trigger PS-exposing procoagulant platelets [43, 44]. The latter is in line with our data and suggests that NETs are not a single agonist but a platform that presents a number of agonists that can promote platelet activation via multiple pathways.

NETs are made up of DNA and proteins (1:1.67 ± 0.26 g, DNA to proteins) [26]. Histones account for 70% of all NET-associated proteins [18, 26, 29, 45]. Previous studies showed the ability of single-strand DNA to bind platelets [46] while double strand DNA can induce platelet aggregation [47, 48]. Degradation of DNA with DNase has been shown to digest NETs and reduce platelet aggregates under flow [30], or platelet adhesion to NETs under static condition [12]. On the other hand, histones are well-established as platelet agonists that can trigger a cascade of platelet responses with defined surface receptors and signalling pathways [18, 29, 45]. Heparin has been reported to bind to histones, thus preventing its binding to platelets [19, 32]. Surprisingly, in this study DNase- and heparin-treated NETs were still capable of aggregating washed platelets and induced expression of P-selectin and active αIIbβ3 to the same extent of untreated NETs. Although DNase and heparin can destabilise the NET structure [9], the presence of freely suspended individual NET components – such as cell free-DNA, histones, and neutrophil proteases – may have greater capacity and exposure to directly bind and activate platelets, as opposed to being restricted on NETs. This presumption is in line with a study that showed DNAse-treatment of NETs resulted in increased coagulation effect [15]. Moreover, nuclear histones have different molecular mass and stoichiometry compared to NET-derived histones [26]. Therefore they may exhibit different biological activity. Indeed, a recent report has found that individual histones and DNA capable of inducing coagulation, but not intact NETs that were released from human neutrophils [49].

Histones are known to induce platelet Syk kinase activation through GPVI, and other tyrosine kinase-linked receptors [29]. We show that inhibition of Syk attenuated NET-induced platelet responses. However, inhibitors of histone receptors on platelets (TLR2, TLR4 and GPVI) did not reduce NET-induced platelet aggregation. Moreover, the sheer magnitude of platelet aggregation response to NETs in washed system precludes a significant contribution of histones which are known to have a tenfold lower platelet aggregation response in washed platelets compared to PRP [29].

Neutrophil granular proteins are a part of NETs and have separately been shown to activate platelets [37, 50]. Neutrophil serine proteases and histones are negatively charged proteins [51] and would be tightly bound to the positively charged DNA backbone of NETs, thus most likely remain bound after isolation procedures [26]. The pre-treatment of NETs with an MPO inhibitor did not significantly reduce NET-induced upregulation of P-selectin, active αIIbβ3, or PS exposure on platelets, suggesting MPO does not play a major role in NET-induced platelet activation which is consistent with previous studies reporting that MPO is not a robust activator of platelets, but only induces partial activation or priming of platelets [37]. On the other hand, inhibiting Cat G resulted in a significant decrease in platelet surface expression of P-selectin, active αIIbβ3 and PS. This suggests Cat G as a molecular mediator of NET-induced platelet activation, and potentially significant contributor to thrombus formation, as previously described [36]. NE is the second most abundant NET-associated proteins after histones [26] and can potentiate Cat G-induced platelet aggregation [52], however in our hands, NE inhibitor did not affect NET-induced platelet responses (data not shown).

As the NET scaffold contains an array of associated proteins, some of which have been independently associated with platelet activation [26, 36, 37, 50, 52–54], a single inhibitor is highly unlikely to completely abrogate NET-induced platelet responses. However, we were interested mainly in clarifying the major NET components, platelet receptors and downstream signalling molecules that mediate NET-induced platelet secretion and aggregation. Inhibition of the tyrosine kinase Syk activity, P2Y_12_ and αIIbβ3 reduced NET-induced platelet aggregation and secretion. While inhibition of NOX1 and TxA2 reduced NET-induced platelet dense granule secretion, but not aggregation.

The role of NETs in initiating thrombosis in vivo has been established in mice models of different diseases [55]. However, the molecular mechanisms that drive NET-induced thrombosis are not well understood. As the recent study by Noubouossie et al., has demonstrated that intact NETs do not directly initiate coagulation [49], we propose that platelets but not coagulation factors are more likely to be the main target of NETs in thrombosis. Our study did not account for the disrupting effect of NETs on endothelium which can also initiate thrombosis [56, 57]. We propose that platelets adhere mainly to NET-derived DNA, then multiple NET-bound proteins induce platelet aggregation and PS exposure which then can propagate coagulation and thrombin generation. In addition to their pivotal role in thrombosis, platelets can also orchestrate inflammation [58]. Therefore, although dismantling NETs may reduce NET-induced thrombosis [59], inhibition of platelet activity may not only reduce thrombosis but also platelet-mediated inflammation. Indeed, Jansen et al., have recently shown that platelet inhibition with clopidogrel was superior to DNase in reducing granulocyte activation, NET formation and acute kidney injury in a renal reperfusion injury mice model [31]. Apart from NETs inducing thrombosis, Cedervall et al. also showed that the use of DNase to disrupt tumour-induced NETs resulted in decreased neutrophil-platelet complexes in the kidney vasculature, along with improved vascular function in tumour-bearing mice [60]. Thus in these contexts NETs can also be considered as scaffold for platelets that drives inflammatory reactions.

## Conclusion

This study showed for the first time that in vitro generated NETs can directly induce marked platelet aggregation. We further identified the molecular pathways activated in platelet responses to NETs. It is important to note that aspirin, a widely used antiplatelet, was not as effective at reducing NET-induced platelet aggregation as ticagrelor or Reopro. Finally, pretreatment of NETs with DNase or heparin did not reduce NET-induced activation or aggregation of human washed platelets. Taken together, we conclude that targeting certain platelet activation pathways rather than NET scaffold has a more profound reduction on NET-induced platelet aggregation. Further in vitro studies are needed to compare the effect of different inhibitors on NET-induced platelet responses in a more complex system such as under flow conditions.

## Additional file

Additional file 1: Supplementary data and figures.ᅟ(PPTX 432 kb)

## Abbreviations

ADP: Adenosine diphosphate
ATP: Adenosine triphosphate
Cat G: Cathepsin G
CTH: Calf thymus histones
Hep: Heparin
MPO: Myeloperoxidase
NETs: Neutrophil extracellular traps
TLR: Toll-like receptor
Tyr: Tyrosine
