# Supplementary material for: Neutrophil extracellular traps induce aggregation of washed human platelets independently of extracellular DNA and histones
Source: Cell Commun Signal. 2018 May 29;16:24. doi: 10.1186/s12964-018-0235-0 (PMC5975482; doi:10.1186/s12964-018-0235-0)
Supplement: Supplementary file 1 — Supplementary data and figures.ᅟ(PPTX 432 kb) [file 12964_2018_235_MOESM1_ESM.pptx]

## Slide 1
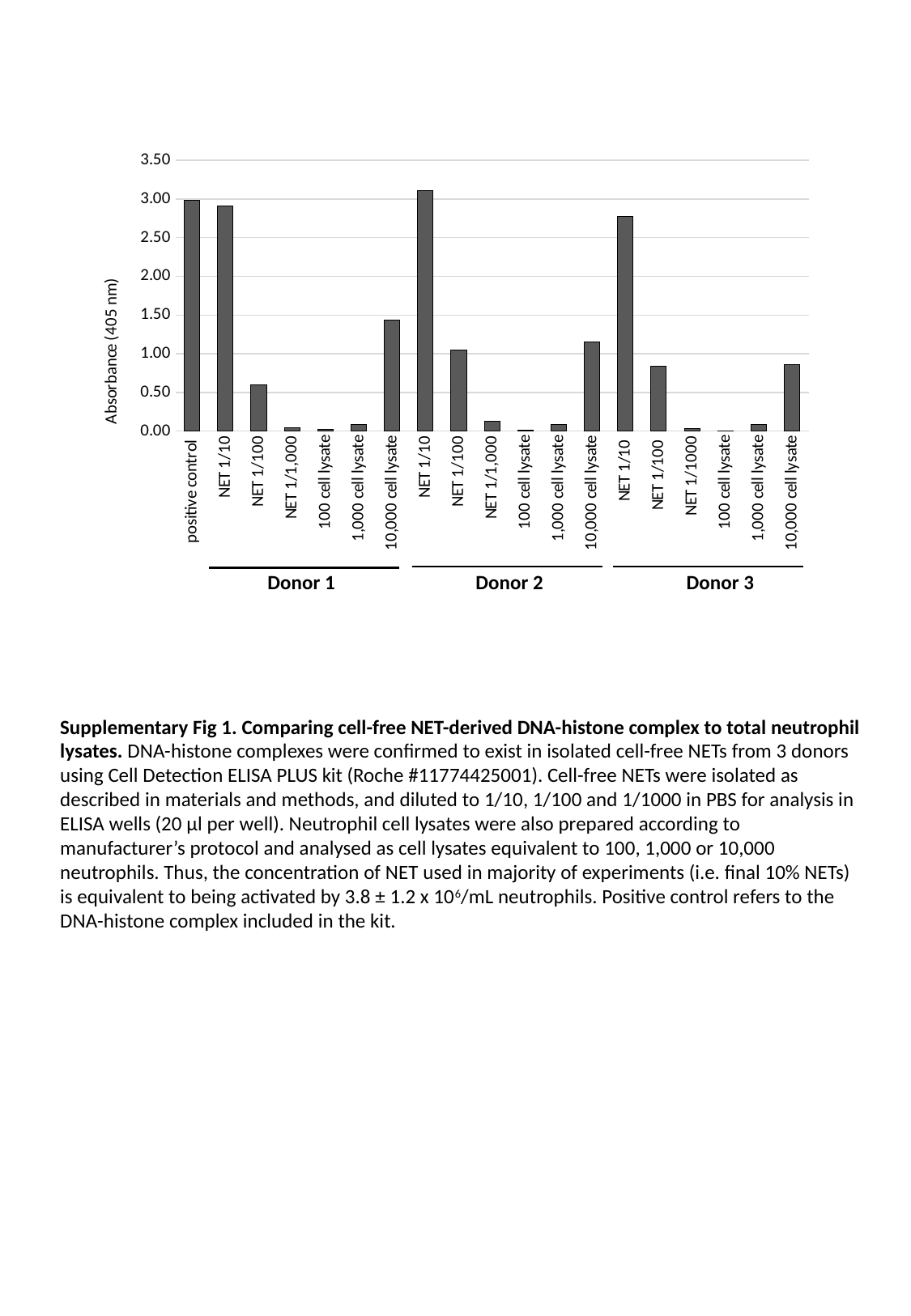

### Chart
| Category | |
|---|---|
| positive control | 2.982 |
| NET 1/10 | 2.9085 |
| NET 1/100 | 0.599 |
| NET 1/1,000 | 0.04050000000000001 |
| 100 cell lysate | 0.018000000000000002 |
| 1,000 cell lysate | 0.089 |
| 10,000 cell lysate | 1.435 |
| NET 1/10 | 3.107 |
| NET 1/100 | 1.045 |
| NET 1/1,000 | 0.122 |
| 100 cell lysate | 0.01150000000000001 |
| 1,000 cell lysate | 0.08099999999999999 |
| 10,000 cell lysate | 1.1545 |
| NET 1/10 | 2.777 |
| NET 1/100 | 0.8340000000000001 |
| NET 1/1000 | 0.03750000000000002 |
| 100 cell lysate | 0.0049999999999999906 |
| 1,000 cell lysate | 0.0815 |
| 10,000 cell lysate | 0.8545 |Donor 2
Donor 1
Donor 3
Supplementary Fig 1. Comparing cell-free NET-derived DNA-histone complex to total neutrophil lysates. DNA-histone complexes were confirmed to exist in isolated cell-free NETs from 3 donors using Cell Detection ELISA PLUS kit (Roche #11774425001). Cell-free NETs were isolated as described in materials and methods, and diluted to 1/10, 1/100 and 1/1000 in PBS for analysis in ELISA wells (20 µl per well). Neutrophil cell lysates were also prepared according to manufacturer’s protocol and analysed as cell lysates equivalent to 100, 1,000 or 10,000 neutrophils. Thus, the concentration of NET used in majority of experiments (i.e. final 10% NETs) is equivalent to being activated by 3.8 ± 1.2 x 10^6^/mL neutrophils. Positive control refers to the DNA-histone complex included in the kit.

## Slide 2
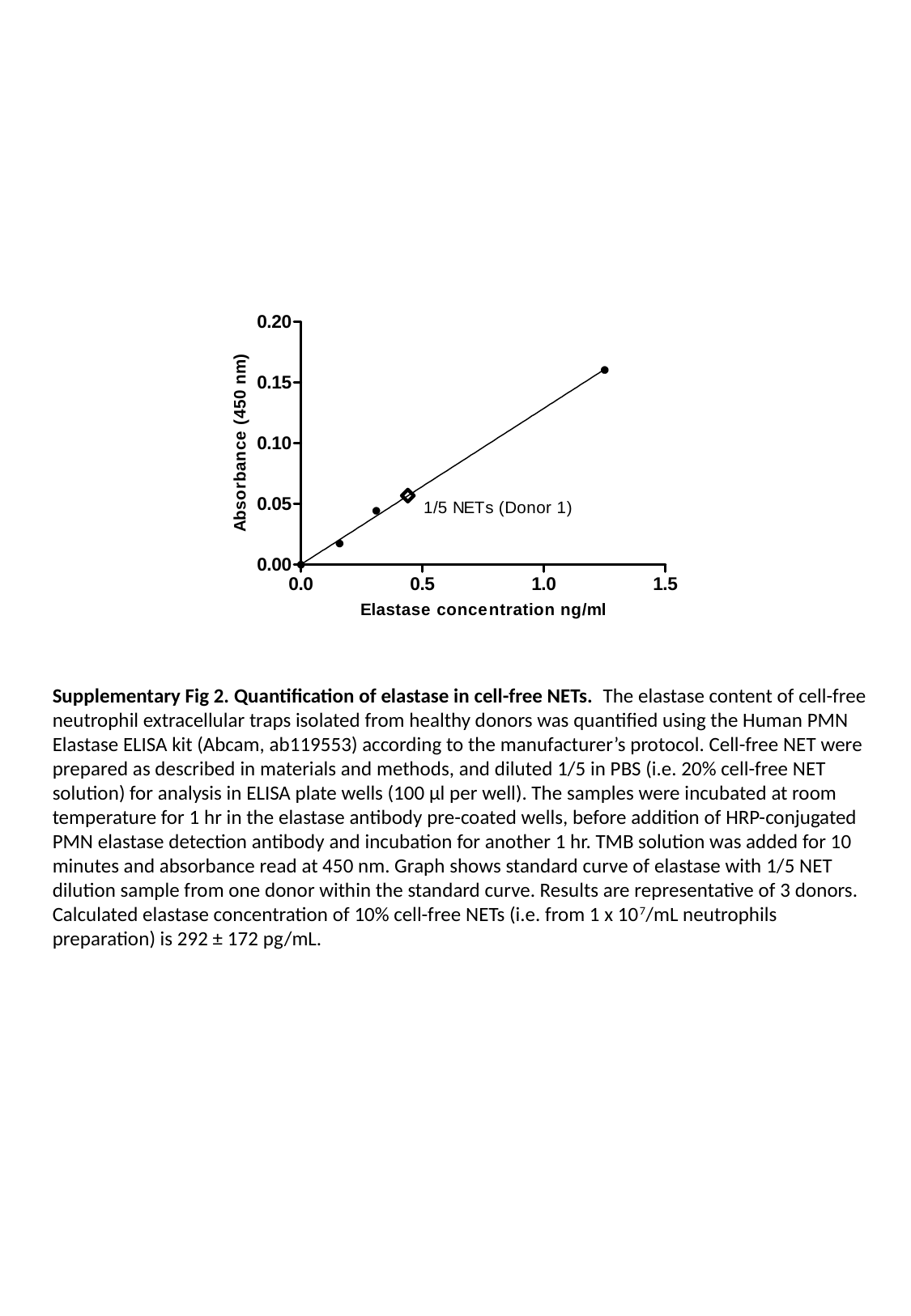

Supplementary Fig 2. Quantification of elastase in cell-free NETs. The elastase content of cell-free neutrophil extracellular traps isolated from healthy donors was quantified using the Human PMN Elastase ELISA kit (Abcam, ab119553) according to the manufacturer’s protocol. Cell-free NET were prepared as described in materials and methods, and diluted 1/5 in PBS (i.e. 20% cell-free NET solution) for analysis in ELISA plate wells (100 µl per well). The samples were incubated at room temperature for 1 hr in the elastase antibody pre-coated wells, before addition of HRP-conjugated PMN elastase detection antibody and incubation for another 1 hr. TMB solution was added for 10 minutes and absorbance read at 450 nm. Graph shows standard curve of elastase with 1/5 NET dilution sample from one donor within the standard curve. Results are representative of 3 donors. Calculated elastase concentration of 10% cell-free NETs (i.e. from 1 x 10^7^/mL neutrophils preparation) is 292 ± 172 pg/mL.

## Slide 3
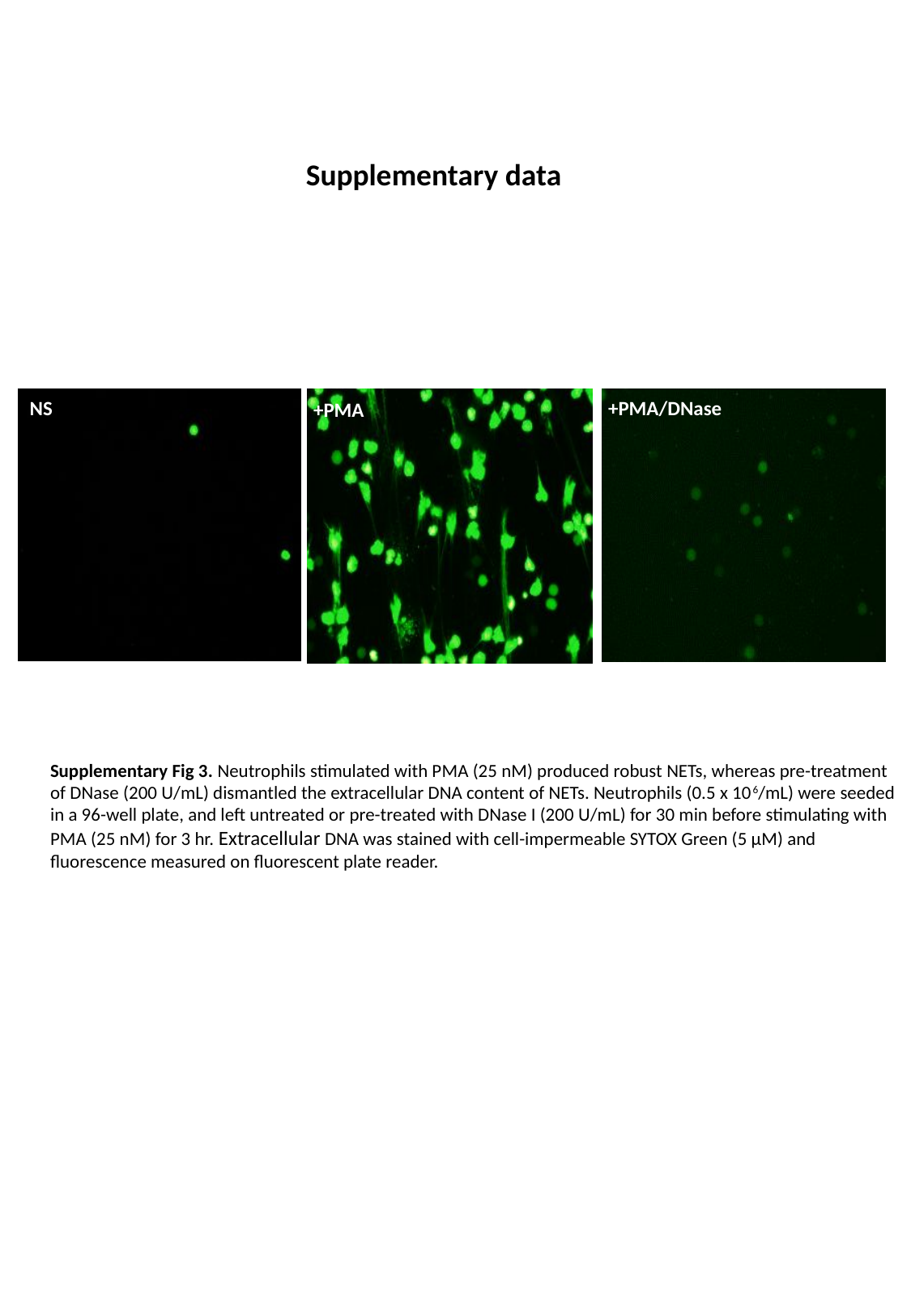

Supplementary data
NS
+PMA/DNase
+PMA
Supplementary Fig 3. Neutrophils stimulated with PMA (25 nM) produced robust NETs, whereas pre-treatment of DNase (200 U/mL) dismantled the extracellular DNA content of NETs. Neutrophils (0.5 x 10^6^/mL) were seeded in a 96-well plate, and left untreated or pre-treated with DNase I (200 U/mL) for 30 min before stimulating with PMA (25 nM) for 3 hr. Extracellular DNA was stained with cell-impermeable SYTOX Green (5 µM) and fluorescence measured on fluorescent plate reader.

## Slide 4
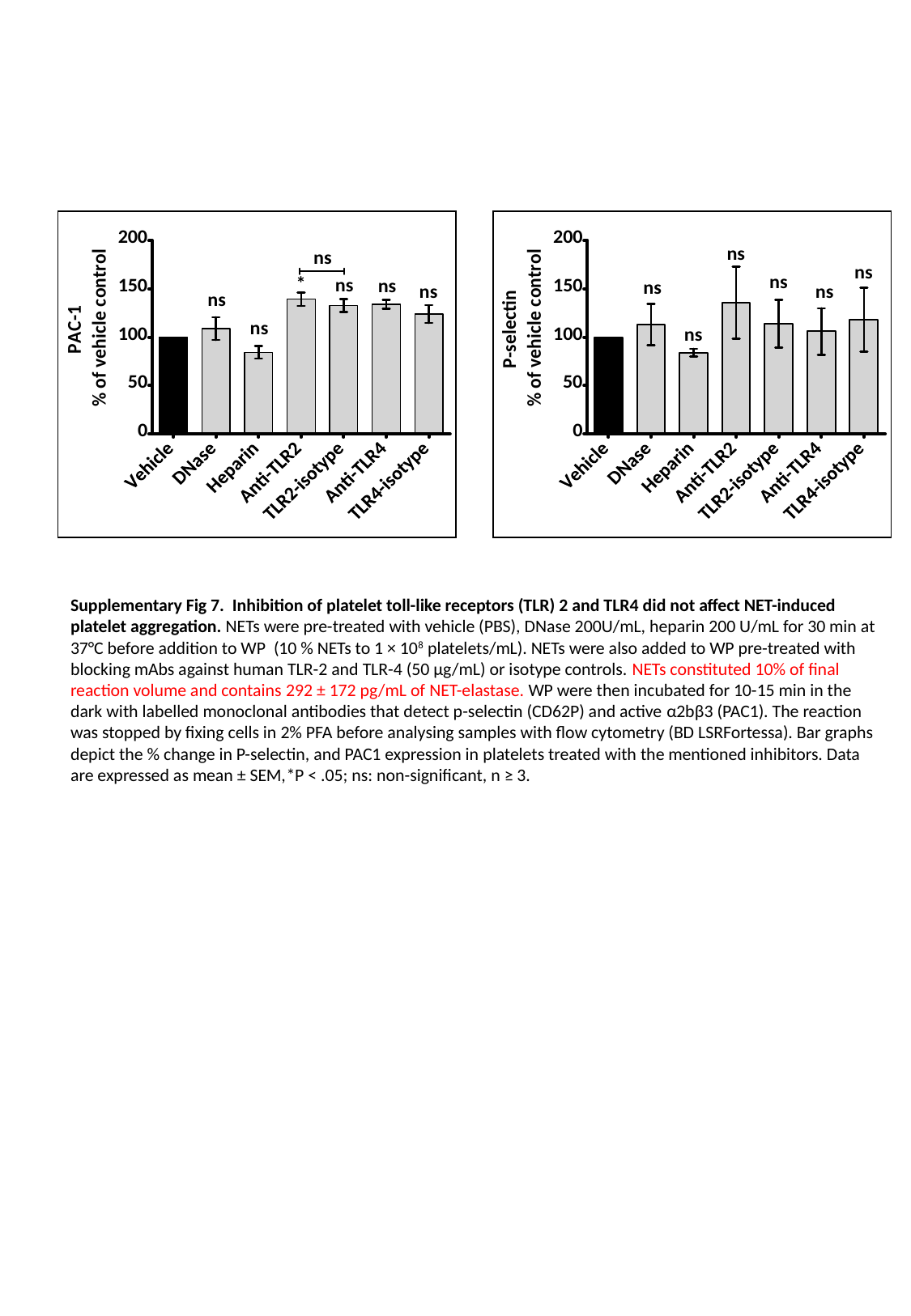

Supplementary Fig 7. Inhibition of platelet toll-like receptors (TLR) 2 and TLR4 did not affect NET-induced platelet aggregation. NETs were pre-treated with vehicle (PBS), DNase 200U/mL, heparin 200 U/mL for 30 min at 37°C before addition to WP (10 % NETs to 1 × 10^8^ platelets/mL). NETs were also added to WP pre-treated with blocking mAbs against human TLR-2 and TLR-4 (50 µg/mL) or isotype controls. NETs constituted 10% of final reaction volume and contains 292 ± 172 pg/mL of NET-elastase. WP were then incubated for 10-15 min in the dark with labelled monoclonal antibodies that detect p-selectin (CD62P) and active α2bβ3 (PAC1). The reaction was stopped by fixing cells in 2% PFA before analysing samples with flow cytometry (BD LSRFortessa). Bar graphs depict the % change in P-selectin, and PAC1 expression in platelets treated with the mentioned inhibitors. Data are expressed as mean ± SEM,*P < .05; ns: non-significant, n ≥ 3.

## Slide 5
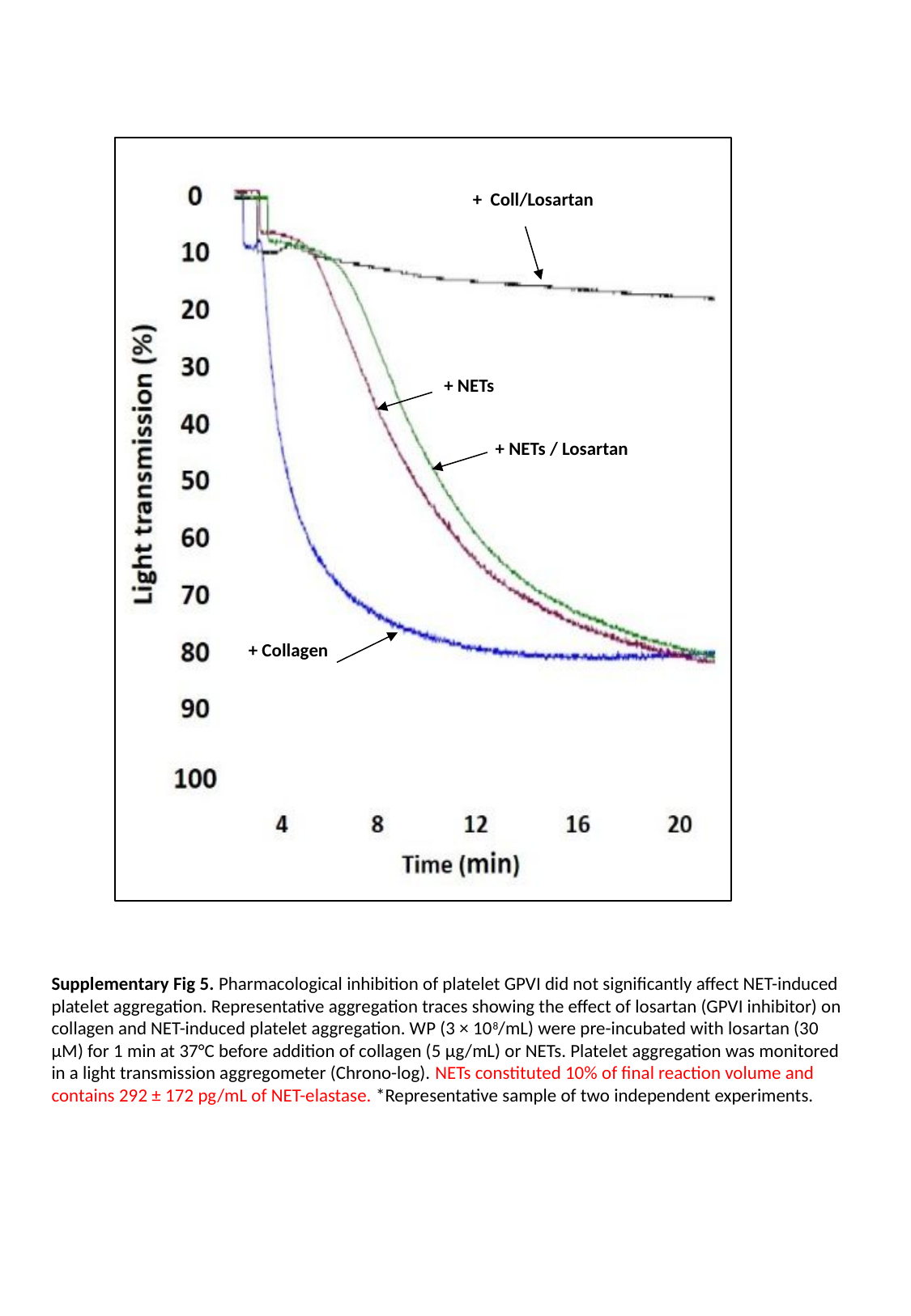

+ Coll/Losartan
+ NETs
+ NETs / Losartan
+ Collagen
Supplementary Fig 5. Pharmacological inhibition of platelet GPVI did not significantly affect NET-induced platelet aggregation. Representative aggregation traces showing the effect of losartan (GPVI inhibitor) on collagen and NET-induced platelet aggregation. WP (3 × 10^8^/mL) were pre-incubated with losartan (30 µM) for 1 min at 37°C before addition of collagen (5 µg/mL) or NETs. Platelet aggregation was monitored in a light transmission aggregometer (Chrono-log). NETs constituted 10% of final reaction volume and contains 292 ± 172 pg/mL of NET-elastase. *Representative sample of two independent experiments.

## Slide 6
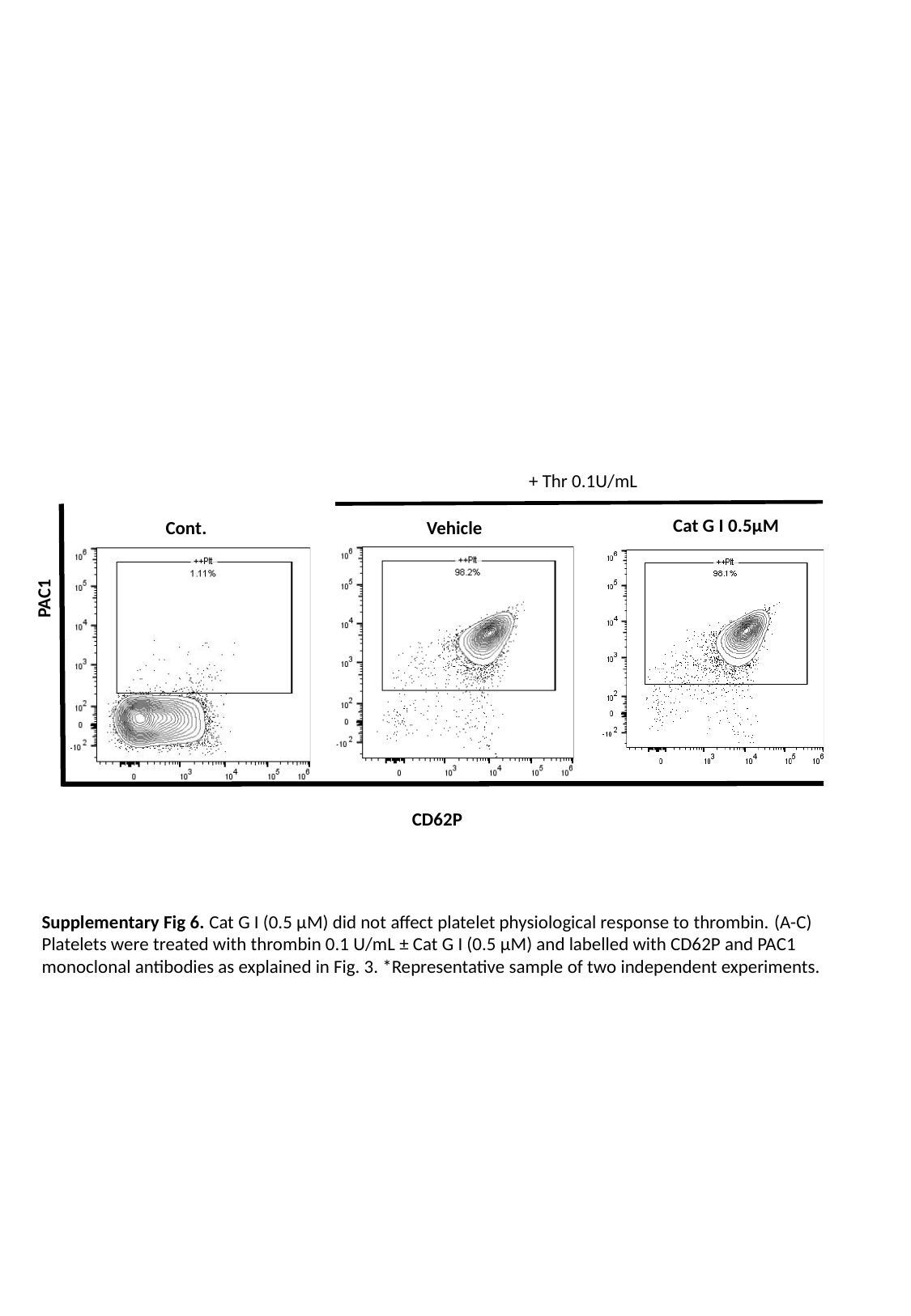

+ Thr 0.1U/mL
Cat G I 0.5µM
Cont.
Vehicle
PAC1
CD62P
Supplementary Fig 6. Cat G I (0.5 µM) did not affect platelet physiological response to thrombin. (A-C) Platelets were treated with thrombin 0.1 U/mL ± Cat G I (0.5 µM) and labelled with CD62P and PAC1 monoclonal antibodies as explained in Fig. 3. *Representative sample of two independent experiments.

## Slide 7
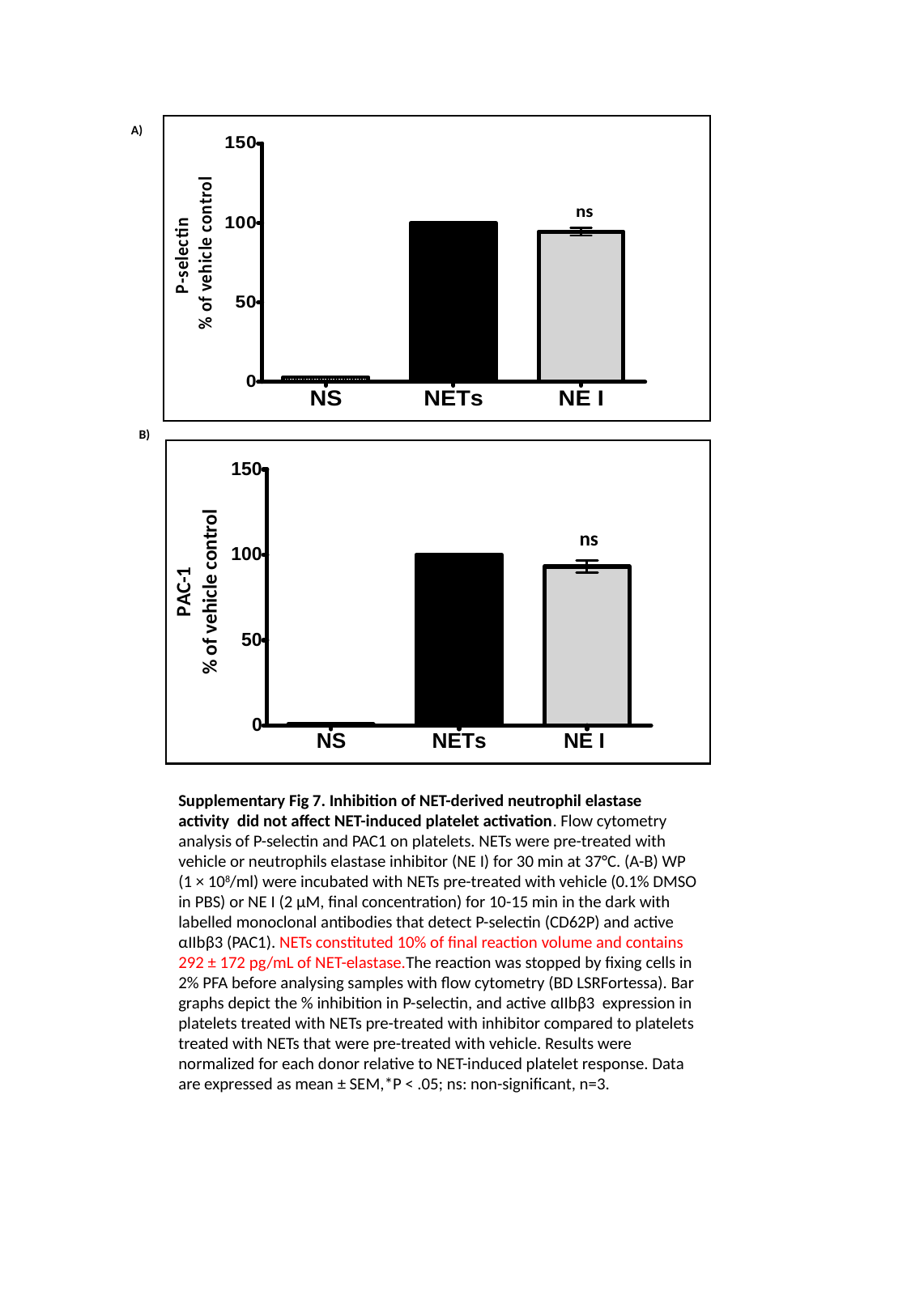

A)
B)
Supplementary Fig 7. Inhibition of NET-derived neutrophil elastase activity did not affect NET-induced platelet activation. Flow cytometry analysis of P-selectin and PAC1 on platelets. NETs were pre-treated with vehicle or neutrophils elastase inhibitor (NE I) for 30 min at 37°C. (A-B) WP (1 × 10^8^/ml) were incubated with NETs pre-treated with vehicle (0.1% DMSO in PBS) or NE I (2 µM, final concentration) for 10-15 min in the dark with labelled monoclonal antibodies that detect P-selectin (CD62P) and active αIIbβ3 (PAC1). NETs constituted 10% of final reaction volume and contains 292 ± 172 pg/mL of NET-elastase.The reaction was stopped by fixing cells in 2% PFA before analysing samples with flow cytometry (BD LSRFortessa). Bar graphs depict the % inhibition in P-selectin, and active αIIbβ3 expression in platelets treated with NETs pre-treated with inhibitor compared to platelets treated with NETs that were pre-treated with vehicle. Results were normalized for each donor relative to NET-induced platelet response. Data are expressed as mean ± SEM,*P < .05; ns: non-significant, n=3.

## Slide 8
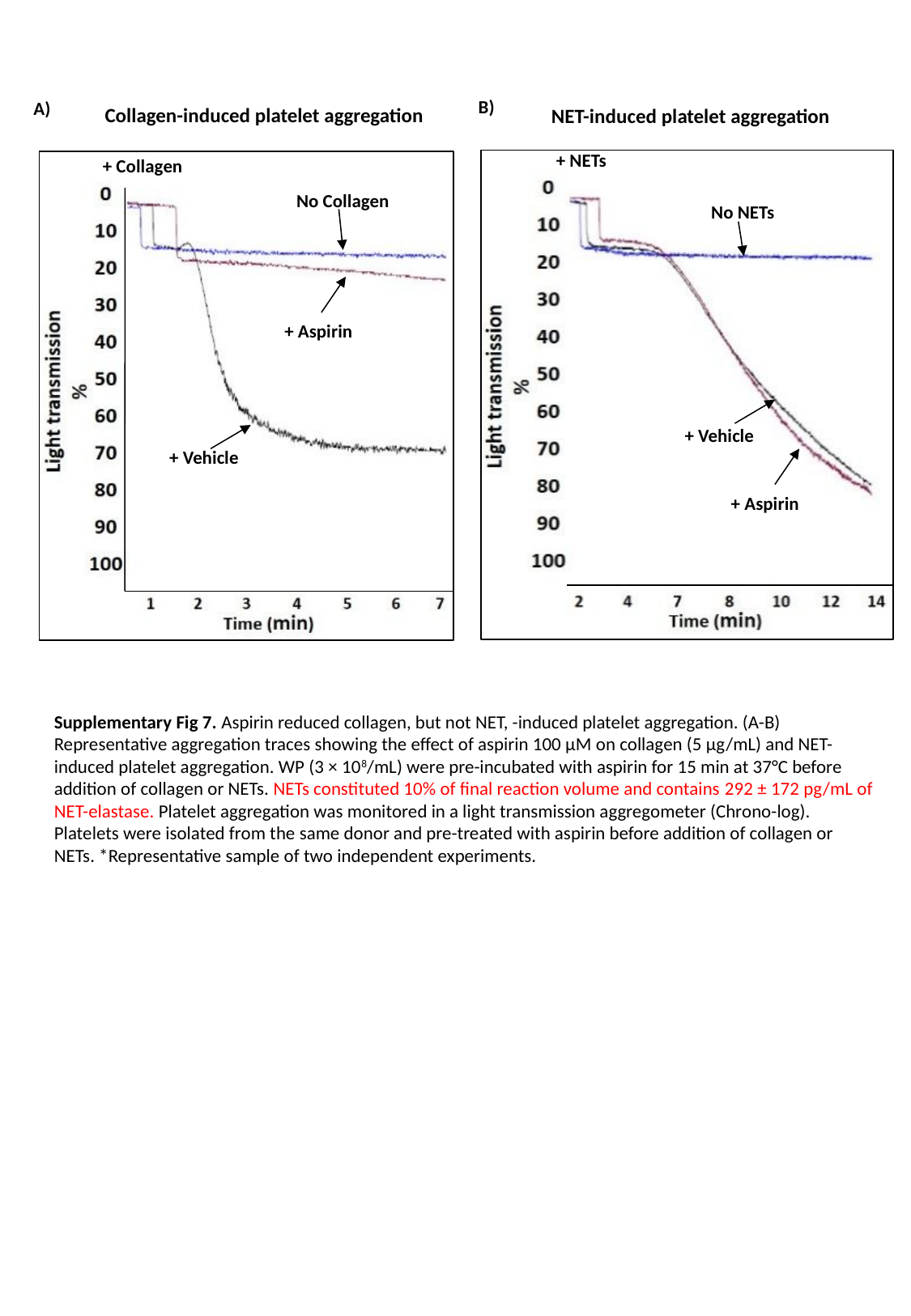

B)
A)
Collagen-induced platelet aggregation
NET-induced platelet aggregation
+ NETs
No NETs
+ Vehicle
+ Aspirin
+ Collagen
 No Collagen
+ Aspirin
+ Vehicle
Supplementary Fig 7. Aspirin reduced collagen, but not NET, -induced platelet aggregation. (A-B) Representative aggregation traces showing the effect of aspirin 100 µM on collagen (5 µg/mL) and NET-induced platelet aggregation. WP (3 × 10^8^/mL) were pre-incubated with aspirin for 15 min at 37°C before addition of collagen or NETs. NETs constituted 10% of final reaction volume and contains 292 ± 172 pg/mL of NET-elastase. Platelet aggregation was monitored in a light transmission aggregometer (Chrono-log). Platelets were isolated from the same donor and pre-treated with aspirin before addition of collagen or NETs. *Representative sample of two independent experiments.
